# Missouri Valley Veterinary Association

**Published:** 1900-03

**Authors:** 


### MISSOURI VALLEY VETERINARY ASSOCIATION.

(Concluded from page 117.)

The President called on Dr. A. T. Peters, of Lincoln, Nebraska, who read a paper on "American *vs.* European Inspection. (See page 140.)

### *Discussion.*

Dr. Forbes: I must say that Dr. Peters has treated the subject in a manner far different from what I had anticipated. What he has said in regard to the meat-inspection service of this country is certainly very flattering to that service. During the last five or six years I think that the system of inspection as carried on by the Bureau of Animal Industry has come to be an almost complete system, and will compare favorably with that of any other country in the world. A great deal has been said about the scientific manner in which meat-inspection is carried on in Germany, but we learn from Dr. Peters' remarks to-night that our system will compare favorably with that in vogue in Germany. I fully believe that the efforts put forth by those in authority in regard to this inspection will ultimately result in a system complete and perfect in every detail, when the mark of the United States Government will be a sufficient guarantee to any country in the world as to the purity and wholesomeness of its meat products.

Dr. Stewart: Permit me to say that Dr. Peters prepared this paper at my suggestion. I had received a communication from the Secretary, Dr. Kelly, stating that there was rather a dearth of papers, and it occurred to me that we could possibly prevail upon Dr. Peters to attend our meeting and prepare a paper for this occasion. I suggested that the paper discuss the views of the people in general, and of those veterinarians who are not in the service, as to the value and quality of the inspection as conducted by the Federal inspectors. That was my idea, rather than comparison with foreign inspection. I think it is beneficial to us "to see oursel's as ithers see us." I thought it would be interesting to the members of the inspecting corps here to know how the people in Lincoln, where they have but a small abattoir, and get most of their meat from South Omaha, look upon the subject of inspection. Dr. Peters travels over the State of Nebraska extensively, and is in touch with other parts of the country, and is certainly in a position to learn the views of the people generally.

However, I would say that he has prepared a paper which is probably more interesting than would be one prepared upon the basis I have indicated. It is gratifying to learn that our inspection bears so favorable a comparison with that of Germany. I am indebted to Dr. Peters for the information that the inspection in that country is not federal, but local. It would certainly seem that where it is under so many authorities it could scarcely be uniform throughout the empire of Germany. It may possibly be extra good in some points and very poor in others, and explains the blunders that have been made, especially in the microscopical work, where they have rural as well as municipal inspection. A layman or man having been but partially instructed in the use of the microscope, is delegated to do rural inspection. His attention is called to the fact that a farmer wants to slaughter a hog and consume it for his own use, and gives a permit for him to do so. The farmer and his friends kill the animal and consume some of the meat sometimes before the inspection is completed. An outbreak of trichinosis occurs, an investigation is made, and it is found they have been eating pork that has not been inspected or if so has been imperfectly inspected and passed.

I wish to allude to a circumstance which occurred in Kansas City perhaps a little more than a year ago. One of the abattoirs in that city was reported in the public press to the effect that the grand jury had found that one of its employés had endeavored to bribe some of the government inspectors to pass condemned meat, and the publication of that article engendered a feeling among the consumers of meat in the surrounding country which made them averse to buying the products of that abattoir. Meats shipped out to towns in the surrounding country were returned with the statement that there was a feeling that that firm was desirous of, if it did not actually engage in, shipping out meats that would not bear inspection. That was an indication of the feeling in the region about Kansas City in regard to inspection. Of course, it needed something of that kind to bring out an expression of sentiment. The inspection certainly is growing in effectiveness, and since hearing this paper I feel much encouraged in regard to it. Dr. Peters has certainly looked upon it with a kindly eye, but I have no doubt whatever that he has drawn a fair comparison, and I believe the publication of that paper will impress our veterinary readers very much in favor of American inspection. Those who have given the matter little attention are ofttimes inclined to look upon the force as a lot of men with government jobs and that it is just a place for a lot of politicians to put their friends.

I believe the publication of this paper in our periodicals might be extended to the lay press. For instance, if the veterinarians of this city would take such an article to their papers and request them to publish it as an extract from the *Veterinary Review*, or the JOURNAL, whichever it may be published in, and the same method followed in other cities, it would in this manner secure an extensive publication. If in St. Joseph it is deemed desirable to cultivate public sentiment along that line, a continual supply of just this sort of articles to your daily press will after a time bring forth substantial fruit in the way of inspection, and is one of the surest and best methods to reach that goal.

Dr. Peters: If I had been near Dr. Stewart at the time he suggested this paper I would have written it somewhat differently. I am very sorry I did not know just what he wanted, but I would beg leave now to say a few words on the subject he has suggested. I think the value of meat-inspection in this country was never more clearly demonstrated than during the controversy over the meat supply to the army during the late unpleasantness. I travel on an average over a thousand miles a month in the State of Nebraska, and if I was accosted once on this subject I was a dozen times during the time to which I have alluded, and I was pleased to see the people defend the federal government. They never believed the accusations which the press made against the federal inspectors. When in 1894 I took the position I now hold it was a common thing to hear such a remark as "Oh, such and such an animal will pass all right." You do not hear any more of that. The people of the State of Nebraska at least have come to the conclusion that they are not going to be cheated, but, on the contrary, they are going to be protected. They were probably, as Dr. Stewart intimated, skeptical at the beginning, and insinuations were made as to the ease with which the inspectors might be bribed. True, an inspector might be bribed occasionally, we cannot help that; but the people have found that the federal government has a check on such things as that, and it can soon be located and rectified. I can assure anyone who is connected with the meat-inspection that he may be proud he is in the service. It is superfluous to say that many things might be suggested which if adopted would benefit the service, but it must be remembered that meat-inspection has been in vogue in this country but a short time, comparatively, and when one has an opportunity of personally comparing the efficiency of the systems in other countries with that of our own, I think the conclusion that we have a superior system is unavoidable.

You gentlemen here read the extracts from Ostertag and other authorities, and you read the comments in our journals, but there are hundreds of men, in the southern part of Germany especially, who have never graduated, but who are appointed, and who supervise the killing of animals and inspect the meat, and in Ostertag's book you will notice that a great many times it says an animal had to be killed, say in a case of choke. The laymen quack will say it is liable to die, and they will kill it, and that meat is used for food, and so with very many other diseases.

Dr. Moore: Dr. Peters' remarks suggest a thought upon which I would like to ask for information. It is a common thing in this country to run across German practitioners who claim to have been practitioners under the German government, whom we know, from their work and what we can see and learn from them, are not qualified men, and I would like to ask if it is a fact that the German government does employ such men.

Dr. Peters: They are employed as inspectors but not by the government. Take the State of Wittenberg for example. Parties in that State occupying public positions similar to county commissioners here appoint Mr. So-and-So an inspector, and he may inspect for trichiniæ only. In Prussia it is necessary to receive instruction for twelve weeks, and I think that is the period at an abattoir, and an examination passed before they are allowed to practise inspection; but they are supposed to call in a veterinarian whenever they think that it is a contagious disease; but these people in a great many instances think they know more than the veterinarians (usually quacks do), and take upon themselves too much responsibility. In the German army and other government positions only qualified men are employed.

Dr. Moore: Another question: Dr. Peters states that it is the common practice of the empiric, for instance, when he is called and finds a case of choke which he thinks is going to prove fatal, to advise the destruction of the animal and its use as food. I would like to know if in such a case any inspection is made by other authorities?

Dr. Peters: Not if he is present before the animal is killed.

Dr. Stewart: I find in Zuill's Friedberger and Fröhner statements relative to diseases of food animals, that in many of the diseased conditions which are likely to destroy life, which are not amenable to treatment, they recommend the destruction of the animal and the use of the carcass for food purposes. For instance, if a cow was suffering from parturient paresis, milk fever, or was paralyzed, we may say moribund, would recommend that it be killed, dressed, and used for food. If a cow were suffering from traumatic pericarditis, in which the pericardial inflammation was of a purulent character, yet the animal was not quite dead, perhaps about to die, certainly could not be relieved by treatment—recommend that the animal be slaughtered and the flesh used for food. And so in many other cases in which in this country the flesh would be condemned as unfit for food. Viewed from this stand-point, American inspection is certainly much superior to German or French inspection. Animals are so abundant in this country, and the price of flesh so low, comparatively, that public sentiment demands its absolute freedom from disease, and would, I believe, often transcend the zeal of the inspector and reject what he passes, because their sentiment is not always founded upon knowledge. I do not know to what extent inspectors generally are governed or influenced by sentiment, but I know that with some it is a potent factor. If they would not like to eat the meat themselves they would not like anybody else to eat it, and put the condemnation mark upon it. Under the circumstance of the cheapness and abundance of meat in this country it seems to me that such a sentiment should be given some play.

Dr. Forbes: I do not know anything about the system of meat-inspection in Germany, but I have had a little experience with it as it is carried on in the city of Glasgow, Scotland, and know of my own personal knowledge that it is quite a common thing for veterinary surgeons when called in cases of parturient paresis, not amenable to treatment, immediately upon arriving at this conclusion they advise the owner to slaughter it and send it into the city for food. The inspector there is a policeman, who acts under the medical health officer, and not one-fourth of them know a diseased condition when they see it. In a case of parturition my employer and myself, after working several hours in a futile effort to relieve the cow, decided that we could do nothing further for her. She was on the point of collapse. The owner was so informed, and he decided to have the animal killed, which was done, the carcass transported to the city, and there placed on the market, so that the more we learn of others the more reason we have to be satisfied with American inspection.

Dr. Goode: Dr. Stewart's remarks remind me of one or two cases that came under my own observation in the city of Chattanooga, Tenn. In one case a very fat steer was brought in, suffering with what afterward proved to be actinomycosis. The matter was noised about in some way, and the captain of police was there and a crowd had congregated around, and the stockyard men telephoned to me to come down and see it. Before I got there the animal had been killed and not bled. It was thrown away at a loss of fifty dollars. They said that even if it was all right they were opposed to using it, for fear something would be said about it and it would damage their business. I said, "You could afford to use it on my say so, couldn't you?" They said, no; they did not care to risk it.

In another case I was called to treat a cow in a case of chronic prolapsus of the rectum. One night it came out, and we could not get it back. The cow was two months after calving and was fat, and I advised the owner to slaughter her for beef, but he said he did not want to do it. I placed an écraseur around the mass and cut it off, and the cow died from the shock, and he lost the value of the cow. These are two cases I know of where the owner incurred a loss just for fear of public opinion.

Dr. Moore: I think we ought to move a vote of thanks to the people of that community.

